# A Five-Decade Perspective on the Evolution of Gelatin and Collagen Technologies and Materials Used in Medical Devices

**DOI:** 10.3390/gels12100896

**Published:** 2026-10-03

**Authors:** Aubrey Woodroof, Gail Naughton, Michelle Montgomery

**Affiliations:** 1 Stedical Scientific Inc., Carlsbad, CA 92009, USA; aubreywoodroof@hotmail.com; 2 Regenemed Associates, Lake Oswego, OR 97034, USA; gnaughton@regenemedassoc.com; 3 GELITA USA, Sioux City, IA 51054, USA

**Keywords:** collagen, gelatin, biomaterials, medical devices, temporary skin substitutes, burn wounds, wound healing, Biobrane, TransCyte, AWBAT, PermeaDerm, regenerative medicine

## Abstract

Collagen and gelatin have been incorporated into medical devices for more than five decades because they combine biological familiarity with practical material versatility. This narrative historical perspective examines how these materials progressed from hemostatic agents, wound coverings, and collagen-stabilized devices toward more deliberately engineered wound-contacting matrices and regenerative biomaterials. The discussion integrates selected peer-reviewed literature with first-hand historical knowledge from authors involved in the development of several technologies. The first part summarizes the material basis for collagen and gelatin use, including their structural differences, biocompatibility, biodegradability, cell interactions, absorbency, processability, and emerging recombinant or animal-free platforms. The second part summarizes selected clinical developments, beginning with collagen fixation chemistry for tissue heart valves and then examining Biobrane, TransCyte, AWBAT, and PermeaDerm as examples of evolving temporary skin-substitute design. Across these technologies, device performance depended not only on the biological material but also on adherence, fluid transport, porosity, transparency, manufacturability, and clinical handling. The available evidence is heterogeneous and includes preclinical studies, clinical comparisons, and first-hand development reports. Future progress will require better material characterization, independent and adequately powered clinical studies, and designs that balance biological function with manufacturability, cost, regulatory clarity, and ease of use.

## 1. Science of Collagen and Gelatin Materials in Regenerative Medicine and Medical Devices

Collagen and gelatin are useful in medical devices because they are closely connected to the extracellular matrix involved in tissue structure and repair. Collagen is the most abundant structural protein in mammals and is present in skin, bone, cartilage, tendon, ligament, blood vessels, and other connective tissues. Its triple-helical structure provides strength and organization, while specific amino acid sequences help cells attach, migrate, and remodel the surrounding matrix [1,2]. Gelatin is produced when native collagen is partially hydrolyzed or thermally denatured. Although that processing disrupts the native collagen structure, it also makes gelatin easier to use in many device formats. It can be processed in water, formed into films or hydrogels, adjusted for viscosity and degradation behavior, and cross-linked when longer residence time or added stability is needed [2,3,4].

This article is a structured narrative historical perspective rather than a systematic or scoping review. PubMed, Scopus, and Google Scholar were searched from database inception through September 2026. The search combined controlled vocabulary, where available, with free-text terms for the materials, medical-device applications, and named technologies addressed in this review. The principal concepts were collagen, gelatin, biomaterial, medical device, tissue heart valve, burn, wound healing, temporary skin substitute, Biobrane, TransCyte, AWBAT, and PermeaDerm. Searches were supplemented by backward citation searching of included publications and by review of patents, books, and first-hand historical reports when these sources documented device composition, manufacturing concepts, design rationale, or development chronology not available in peer-reviewed articles.

Sources were eligible when they described material composition or processing, device development, design rationale, preclinical performance, clinical use, or clinical outcomes relevant to the technologies discussed. English-language peer-reviewed original studies and reviews were prioritized. Patents, books, and author-derived historical records were retained only when they contributed primary technical or chronological information and were identified as such in the text. Sources were excluded when they addressed collagen or gelatin solely as nutritional ingredients or cosmetic materials without a medical-device application, discussed unrelated wound products without a relevant comparator, duplicated an included report, or provided insufficient information to support the historical or technical discussion. Titles and abstracts were reviewed for relevance, followed by full-text assessment of potentially eligible sources. Literature selection was iterative and was designed to support a selective, interpretive historical analysis rather than an exhaustive systematic or scoping review. Accordingly, formal systematic-review procedures, including duplicate independent screening, risk-of-bias scoring, meta-analysis, and PRISMA-based study-flow reporting, were not applied. To maintain transparency while remaining consistent with the narrative-review design, the resulting evidence is described and interpreted throughout the manuscript according to study type, reporting quality, and methodological limitations rather than treated as a uniform body of evidence.

### 1.1. Scientific Basis for Collagen and Gelatin as Regenerative Biomaterials

The appeal of collagen-based materials is not simply that collagen is a natural material. It is that collagen resembles a structure the body already uses. In tissues, collagen fibrils provide mechanical reinforcement and create a framework that cells can recognize and remodel. In medical applications, purified collagen has been processed into sponges, membranes, films, powders, injectable gels, composites, and porous scaffolds. These forms have been used or investigated for wound management, hemostasis, guided tissue regeneration, bone and cartilage repair, dermal matrices, drug delivery, vaccine stabilization, and tissue-engineered constructs. In each case, the material is being asked to provide temporary support, protection, or stabilization while the patient’s own repair process begins, or while a biologically active product must maintain potency during storage and handling [1,3,5,6].

Gelatin offers a related, and often more practical, option for device design. Its biodegradability, biocompatibility, and processability make gelatin a useful scaffold that can support cellular proliferation and migration, which are critical to the healing process [7,8]. Because it is denatured collagen, it does not behave exactly like native collagen fibrils. For many wound-contacting applications, however, that difference can be useful. Gelatin can dissolve or swell in water, form thermoreversible gels, be cast into films, freeze-dried into porous sponges, electrospun into fibers, converted into microparticles, or chemically modified to create materials such as gelatin methacryloyl. That processing flexibility matters for temporary skin substitutes and other wound interfaces, where the material must contact an irregular tissue surface, manage wound fluid, and still be manufactured at a practical scale [2,4,9].

Native collagen and gelatin share an amino acid composition but differ substantially in higher-order structure and material behavior. Collagen retains its characteristic triple-helical molecules and can assemble into fibrillar structures that provide tensile reinforcement and biologically recognizable binding sites. Gelatin is produced by partial hydrolysis and thermal or chemical denaturation of collagen, which disrupts the native triple helix and produces a heterogeneous distribution of soluble polypeptide chains. Below its gelation temperature, gelatin can partially re-form triple-helical junction zones and generate a thermoreversible physical gel; however, this network is generally weaker and less stable at physiological temperature than native collagen fibrils unless it is chemically modified, blended, or cross-linked. Both materials are susceptible to enzymatic degradation, but the rate depends strongly on source, molecular-weight distribution, processing, porosity, and cross-link density. Denaturation can expose some cell-interactive sequences while altering others, so biological activity should not be assumed to be equivalent across collagen and gelatin formulations. Accordingly, quantitative values for strength, swelling, degradation, or cell response are most meaningful when reported for a defined material and test method.

### 1.2. Special Properties of Gelatin Relevant to Regenerative Medicine and Medical Devices

**Biocompatibility and biodegradability.** Gelatin is a biodegradable collagen-derived material that can be broken down by hydrolysis and enzymatic activity, allowing it to serve as a temporary rather than permanent matrix [2,3,4]. **Cell interaction.** Depending on its source and processing history, gelatin can retain sequences that support cell attachment and spreading [1,2,3]. **Hemostatic and absorbent behavior.** Porous gelatin matrices absorb blood and wound fluids and provide a local structure in which clotting components can accumulate [1,3,4]. **Manufacturing flexibility.** Gelatin can be processed into films, foams, sponges, fibers, particles, hydrogels, coatings, and composites [2,4,9]. **Tunable properties.** Gel strength, viscosity, swelling, porosity, dissolution, and degradation depend on source, molecular-weight distribution, concentration, drying method, and cross-linking strategy [2,4,9]. Gelatin-based materials can also be engineered to provide antibacterial or anti-inflammatory activity through cross-linking design and incorporation of functional components [8]. **Carrier function.** Gelatin can also carry drugs, antimicrobial agents, growth factors, cells, or extracellular-matrix components, with formulation and cross-linking used to influence release [2,9].

### 1.3. Historical and Current Uses of Collagen Proteins in the Medical Space

The medical use of collagen proteins has developed over many decades, beginning with applications that were practical and clinically direct. Absorbable gelatin sponges helped with hemostasis, collagen dressings were used in wound care, collagen membranes supported guided tissue and bone regeneration, and collagen-based dermal substitutes, plugs, fillers, and coatings were developed for a range of surgical or device needs. These applications remain important because they illustrate the central value of collagen and gelatin in medicine: both materials can provide a temporary, biologically compatible interface while healing progresses [1,3].

The wider field also includes absorbable gelatin hemostats, collagen membranes used in guided bone regeneration, injectable bovine collagen fillers and later human bioengineered collagen products [10], and dermal or epidermal replacement technologies such as Integra, AlloDerm, Apligraf, Dermagraft, MatriDerm, and Suprathel. These products differ substantially in composition, cellular content, permanence, mechanism of action, and clinical indication. They are included here to position the selected technologies within the broader medical-device landscape; detailed comparison is outside the scope of this historical perspective.

Animal-derived collagen and gelatin also require attention to source, purification, residual processing agents, and patient-specific hypersensitivity. Bovine collagen products have historically been associated with hypersensitivity in a small subset of recipients, and source qualification and controls for transmissible spongiform encephalopathy risk are important for bovine-derived materials. Gelatin can likewise act as an allergen in susceptible individuals when present in vaccines, hemostatic agents, or other medical products. These risks do not preclude clinical use, but they reinforce the need for appropriate sourcing, manufacturing controls, labeling, and clinical vigilance.

More recent work has made these materials more engineered and more specific to the intended tissue response. Examples include mineralized collagen scaffolds for bone repair, collagen-glycosaminoglycan matrices for dermal regeneration, gelatin hydrogels for cell delivery, gelatin microparticles for sustained release of growth factors, gelatin or collagen-derived stabilizers for vaccine formulations, and gelatin-based bioinks for three-dimensional bioprinting. Collagen and gelatin are also frequently combined with hyaluronic acid, alginate, chitosan, synthetic polymers, calcium phosphates, ceramics, or bioactive glass. These combinations are used to adjust strength, degradation behavior, fluid handling, biological response, stabilization of sensitive biological components, and manufacturability for a particular medical application [2,4,5,6,9].

Outside of wound-contacting devices, gelatin and collagen-derived peptides also have a medical role as stabilizing excipients in some vaccine formulations. In that setting, the material is not intended to function as a scaffold for tissue repair. Instead, it helps protect sensitive viral or protein-based vaccine components from stresses such as freeze-drying, heat exposure, agitation, and interaction with container surfaces, thereby helping maintain potency during storage, transport, and administration [6].

Although gelatin is an effective stabilizer, rare immediate hypersensitivity reactions to gelatin-containing vaccines have been reported, particularly in individuals with pre-existing gelatin-specific IgE. This known limitation should be considered separately from animal-source and transmissible spongiform encephalopathy controls, which address raw-material origin and manufacturing risk rather than allergic sensitization.

For wound care and burn management, these material properties become especially important. A wound-contacting material must do more than cover the surface. It must stay in close contact with the wound bed, permit the right degree of fluid movement, protect the tissue, and support the early events involved in re-epithelialization and granulation tissue formation. In hemostatic devices, gelatin’s absorbency and porous structure help concentrate blood and clotting components locally. In tissue engineering, collagen and gelatin are useful because they give cells a recognizable matrix while still allowing the material to be modified for the requirements of a specific tissue or device [1,3,4].

### 1.4. Evolution of Collagen-Based Materials: From Extracted Animal Collagen to Engineered Matrices

The evolution of collagen in medical applications can be understood as a movement from simple biologic coverings and absorbable hemostatic materials to structural scaffolds, dermal substitutes, and more engineered, application-specific matrices. This history is directly relevant to temporary skin substitutes and gelatin-coated wound interfaces. These products rely on collagen-derived biology, but their usefulness also depends on design features that are easy to underestimate: adherence, porosity, fluid handling, transparency, strength, storage, and ease of use [1,2,3,5].

Historically, medical collagen has most often been obtained from animal tissues, including bovine tendon, bovine hide, porcine skin, and fish sources. These materials made it possible to develop absorbable hemostats, wound dressings, surgical matrices, and tissue-regeneration scaffolds. As the field matured, the focus shifted from simply extracting collagen to controlling how the material performs. Improvements in purification, cross-linking, freeze-drying, mineralization, and surface modification have all helped make collagen-based products more predictable and better suited to specific clinical applications [1,3,5].

This progression also changes how these materials should be evaluated. A dressing, scaffold, or coating is not merely a passive covering. It can influence fluid handling, cell attachment, inflammation, vascularization, and remodeling. For temporary wound coverage, the material may need softness, adherence, moisture control, and barrier function. For bone regeneration, the priorities may shift toward interconnected porosity, mineral content, and mechanical stability. Collagen and gelatin are useful across these different settings because they provide a biologically familiar starting point that can be adapted through processing and device design [1,3,4,5].

### 1.5. Recombinant and Animal-Free Collagen: Emerging Direction for Regenerative Medicine

One additional development deserves attention: recombinant, animal-free collagen and collagen-like proteins. Animal-derived collagen remains widely used and clinically important, but it can present practical challenges, including source variability, batch-to-batch differences, possible residual impurities, religious or dietary considerations, and theoretical concerns related to animal-derived pathogens. Recombinant approaches are being developed to address some of these limitations by producing defined collagen sequences in controlled expression systems [11,12,13,14].

Recombinant collagen can be produced in microbial, yeast, plant, insect, mammalian, or transgenic systems, depending on the target structure and required post-translational modifications. For human collagen, hydroxylation of proline and lysine residues is important for triple-helix stability, so the choice of expression system and enzyme co-expression strategy can be critical. Recombinant technology also makes it possible to design collagen fragments, full-length collagen chains, gelatin-like proteins, or fusion proteins with selected cell-binding, degradation, or cross-linking motifs [11,12].

In regenerative medicine, recombinant collagen is being explored for wound repair, dermal regeneration, corneal repair, cartilage and bone applications, drug delivery, and biofabrication. The potential advantages are straightforward: animal-free sourcing, defined composition, improved consistency, and the ability to engineer selected biological or material features. The practical questions are just as important. These materials still need to be scalable, cost-effective, structurally appropriate, mechanically useful, biologically active, and acceptable within medical devices or biologic regulatory pathways. For that reason, recombinant collagen should be viewed as an important emerging option rather than a simple one-for-one replacement for established animal-derived collagen materials [11,12,13,14].

### 1.6. Relevance to Advanced Gelatin Materials for Regenerative Medicine

In the context of this manuscript, gelatin is most important because it links collagen-derived biology with practical device design. It can function as a wound-contacting surface, hemostatic matrix, degradable scaffold, hydrogel precursor, coating, or delivery vehicle. Its collagen origin gives it biological relevance, while its solubility and processability make it easier to incorporate into device formats that must be manufactured, stored, handled, and applied consistently. Future gelatin and collagen-based regenerative materials will likely continue to move toward more controlled, reproducible, and application-specific designs, including recombinant or animal-free platforms when defined composition and supply consistency are important [2,4,11,12].

The following section illustrates how these material principles were translated into clinical technologies over approximately five decades, with emphasis on temporary skin substitutes and related collagen- or gelatin-containing wound interfaces.

## 2. Historical Perspective on Selected Collagen- and Gelatin-Containing Medical Technologies

This section presents a selective historical perspective on two related lines of medical-device development: chemical stabilization of collagen-rich tissue for bioprosthetic heart valves and the subsequent evolution of collagen- or gelatin-containing temporary skin substitutes. Several authors participated directly in portions of this development history. First-hand knowledge is used to clarify design rationale and chronology, while statements regarding safety or clinical performance are attributed to the published evidence. The two lines are therefore presented as parallel examples of how collagen chemistry and device engineering were adapted to different clinical needs, rather than as a single continuous technological pathway.

For interpretive clarity, the evidence in this section is described according to study design: material characterization or in vitro investigation; animal or other preclinical study; single-patient clinical report; uncontrolled clinical series; retrospective comparative study; or prospective randomized comparative trial. These categories are not treated as equivalent. Statements regarding clinical benefit are limited to the populations, comparators, outcomes, and follow-up reported in the underlying studies, and mechanistic explanations are distinguished from directly demonstrated effects.

### 2.1. Early Collagen Fixation Technologies for Tissue Heart Valves

The development of these collagen-based medical technologies began with life-sustaining and life-supporting tissue heart valves. From 1970 to 1975, purified glutaraldehyde (GA) and formaldehyde (FA) solutions were developed for the fixation and stabilization of bovine pericardial and porcine aortic tissue. GA became preferred because it forms stable inter- and intramolecular cross-links in collagen and elastin, thereby improving mechanical strength, durability, and biocompatibility. FA is less effective than GA in forming stable cross-links and is often used in combination with GA, or after fixation, to support sterility requirements [15]. Although glutaraldehyde fixation decreases the antigenicity of bioprosthetic heart valves, it does not eliminate it [16].

The heart-valve work and the later skin-substitute program addressed different clinical problems and should not be interpreted as a direct product lineage. Their connection is instead methodological: in each case, performance depended on controlling the interaction between collagen-derived biological material, chemical stabilization, and a supporting device structure. The following sections therefore treat these developments as parallel historical examples.

### 2.2. Biobrane: Development of a Gelatin-Coated Biosynthetic Skin Substitute

According to the first-hand historical account of co-author Aubrey Woodroof, the development program that ultimately led to Biobrane began in June 1970, when Robert H. Bartlett asked Woodroof to investigate a biosynthetic alternative to frozen cadaveric allograft that could provide temporary burn-wound coverage at lower cost. This practical clinical objective initiated a series of material and design investigations that contributed to the development of gelatin-coated biosynthetic skin substitutes over the following five decades.

Early animal studies conducted during this development program demonstrated the importance of the gelatin coating used in Biobrane [17,18,19,20,21,22]. In comparative wound models, Biobrane initially adhered less strongly than allograft or xenograft, but by 72 h it demonstrated significantly greater and more consistent adherence than either biological dressing. These findings supported the concept that a synthetic support coated with collagen-derived material could interact effectively with the wound bed and provide temporary coverage during the early healing period [18,19,20,21,22].

Biobrane consists of a silicone film bonded to a knitted nylon mesh and coated with a high-molecular-weight, high-Bloom porcine-skin gelatin [23]. Historical molecular-sieve chromatography identified fractions with approximate molecular masses of 95, 190, and 285 kDa, corresponding to α-, β-, and γ-chain-sized species. The available records do not establish the exact Bloom value, gelatin process type, isoelectric range, coating density, or complete manufacturing specifications, and these details should not be inferred from the collagen source. The gelatin was covalently coupled to the substrate using proprietary chemistry intended to retain the coating at the wound interface. Quantitative effects of the coupling process on swelling, degradation, coating retention, or adherence were not reported. The initial design did not contain pores; pores were added later to permit wound-fluid egress and help maintain a moist, but not overly wet, wound environment. Differences in the knitted nylon structure and denier were also used to tailor handling and adherence for different wound applications [23].

The related patent describes surface activation and coupling concepts for attaching biological components to flexible substrates, including fabric–elastomer membrane matrices intended for wound-contacting applications [17]. It is cited here for design and manufacturing concepts, not as the sole evidence for biological performance. The animal adherence findings discussed above are supported by the peer-reviewed studies [18,19,20,21,22].

### 2.3. Clinical Validation of Biobrane in Burn Wound Management

Clinical development addressed a practical problem in the management of extensive full-thickness burns: after early eschar excision, temporary coverage may be needed until autografting can be completed. Such a dressing must protect the wound, limit desiccation and heat loss, permit fluid management, and remain closely apposed to the wound bed. The gelatin coating was intended to support formation of a fibrin-rich interface between the dressing and the wound. In a multicenter, within-patient comparison, 71 patients received both Biobrane and frozen cadaver allograft over freshly excised full-thickness burn sites [24]. Mean burn size was 35 ± 20% of total body surface area, mean full-thickness burn size was 28 ± 20%, and the temporary coverings remained in place for a mean of 10.2 ± 6.7 days. The investigators found no significant between-treatment differences in dressing changes, area replaced, purulence, autograft take, or final outcome, and reported no complications attributable to either covering [24].

Later clinical experience clarified both the usefulness and limitations of Biobrane. Greenwood and colleagues described Biobrane as relatively inexpensive, easy to store, apply, and secure, and reliable when used according to established guidelines [25]. They also emphasized that appropriate patient and wound selection are important, particularly when Biobrane is used beyond superficial or partial-thickness burn indications [25]. This clinical experience reinforced the value of Biobrane while also identifying opportunities for later design improvements in porosity, adherence, and fluid management.

A five-year retrospective cohort study later compared Biobrane with cadaveric allograft for temporary coverage of upper-extremity burns [26]. The analysis included 45 patients: 30 treated with Biobrane and 15 with allograft. Procedure time was lower with Biobrane than with allograft (21.12 versus 54.78 min per percentage of total body surface area excised; *p* = 0.02), as was the reported cost measure (1.30 versus 2.35 dollars per minute per percentage of total body surface area excised; *p* = 0.002). Each group had two revisions before definitive autografting. As a retrospective, nonrandomized analysis with unequal group sizes, the study remains susceptible to baseline and selection differences. The authors suggested that the ease of Biobrane application might also make it useful when rapid temporary coverage is required; proposed uses in transport, military, or mass-casualty settings were not evaluated directly in the study [26].

The Biobrane evidence summarized here includes early animal adherence studies, a multicenter within-patient clinical comparison, practice-based clinical experience, and a later retrospective comparative cohort [18,19,20,21,22,23,24,25,26]. The within-patient comparison provides direct comparative clinical evidence for temporary coverage of excised burns, whereas the retrospective cohort is more vulnerable to selection and baseline differences. Neither design establishes that every reported outcome will generalize to other burn depths, anatomical sites, or care settings.

### 2.4. TransCyte: Biologically Active Wound Coverage Using Fibroblast-Derived Matrix

In the early 1990s, the field moved from passive wound coverage toward biologically active wound interfaces. Building on Biobrane as a burn dressing platform, Naughton and colleagues seeded the mesh with neonatal human fibroblasts established through master and working cell banks [27]. As the cells proliferated, they secreted extracellular matrix proteins, glycosaminoglycans, and growth factors associated with granulation tissue formation and epithelialization [27]. The resulting product was initially named Dermagraft-TC and later renamed TransCyte after FDA approval. Clinical studies evaluated both viable and nonviable versions in deep partial- and full-thickness wounds, and the nonviable product was ultimately favored in the acute burn setting [27,28,29].

Noordenbos and colleagues conducted a prospective randomized comparison using paired wound sites in 14 patients [30]. Mean time to 90% epithelialization was 11.14 days with TransCyte and 18.14 days with silver sulfadiazine (*p* = 0.002). A subsequent noncomparative evaluation included 18 additional patients; no infection was reported among the 32 TransCyte-treated wound sites across both parts of the study. In the randomized group, scars were assessed at 3, 6, and 12 months with the Vancouver Scar Scale. Scores favored TransCyte-treated sites at 3 and 6 months (*p* < 0.001) and at 12 months (*p* = 0.006) [30]. In patients with large-surface-area mid-dermal facial burns, TransCyte treatment was associated with significantly faster healing, reduced pain, and decreased patient-care time [31]. The publication did not report dispersion for the epithelialization means in the available summary, so none is inferred here.

Kumar and colleagues randomized 58 partial-thickness burn wound sites in 33 children to TransCyte (*n* = 20), Biobrane (*n* = 17), or Silvazine (*n* = 21) [32]. Mean time to at least 90% re-epithelialization was 7.5, 9.5, and 11.2 days, respectively; the overall comparison was statistically significant (*p* < 0.001). Autografting was required for 1 of 20 TransCyte sites, 3 of 17 Biobrane sites, and 5 of 21 Silvazine sites. TransCyte and Biobrane also required fewer dressing changes than Silvazine, with the fewest changes reported for TransCyte [32]. The trial supports a comparative clinical advantage within this pediatric cohort, but it does not establish the reasons for TransCyte’s later commercial withdrawal. Explanations related to manufacturing cost and reimbursement reflect historical author perspective and are not findings of the trial.

TransCyte is supported by prospective randomized comparisons in small patient cohorts, together with noncomparative clinical observations and later review evidence [27,28,29,30,31,32]. The randomized studies support comparative conclusions for the specific burn populations and endpoints examined, but their sample sizes, wound-site allocation methods, and study-era practices limit broader inference. The noncomparative observations should be interpreted as supportive clinical experience rather than evidence of comparative efficacy.

### 2.5. AWBAT: Improving Porosity, Adherence, and Antimicrobial Permeability

AWBAT (Advanced Wound Biosynthetic Alternative Tissue) was developed after Biobrane with changes to the nylon structure, pore pattern, continuity of the silicone–nylon membrane, and biological-coating process [33]. The covalent coupling agents used in the earlier Biobrane process were removed as a manufacturing-safety and process-design decision. The published reports do not provide comparative quantitative data showing how this change affected coating retention, swelling, degradation, handling, or wound adherence; accordingly, it is presented here as design rationale rather than demonstrated superiority. The revised pore architecture was intended to reduce fluid accumulation and permit diffusion of water-soluble antimicrobial agents [33,34]. Limited donor-site experience reported no infections and shorter healing with AWBAT than with Acticoat, but the small sample and limited reporting constrain interpretation [33].

The AWBAT evidence presented here is predominantly developmental and preclinical, supplemented by limited donor-site observations [33,34]. The available reports support the stated design rationale and document feasibility, but they do not provide adequately powered randomized comparative evidence of clinical efficacy. AWBAT should therefore be regarded as a promising design iteration for which comparative clinical performance remains preliminary.

### 2.6. PermeaDerm: Variable Porosity and Next-Generation Wound Matrix Design

PermeaDerm extended the concept of controllable porosity by allowing clinicians to adjust moisture balance according to wound needs [34]. Rather than relying on fixed open pores, PermeaDerm prototypes used slit patterns in the silicone layer to help minimize desiccation while limiting fluid accumulation [34]. Several prototypes were evaluated (Table 1). PermeaDerm B and C differed in slit density; PermeaDerm T incorporated native collagen with a gelatin/aloe biological coating; and PermeaDerm AS and AS-2 incorporated anti-scar compounds in the silicone layer, the biological coating, or both [34]. Physical testing was performed with PermeaDerm B or C before biological coatings were added, and sterilized prototypes were subsequently evaluated for safety and performance [34].

### 2.7. Preclinical Safety, Adherence, and Cellular Response of PermeaDerm

Preclinical evaluation compared the topical tissue response and potential systemic effects of PermeaDerm with Biobrane in a two-week rat model. Full-thickness dermal wounds were covered with either PermeaDerm or Biobrane, and wound sites and selected organs were examined grossly and histologically on day 14. Wound sections were evaluated for crust formation, epidermal hyperplasia, re-epithelialization, granulation tissue maturity, necrosis, inflammation, foreign material, and hemorrhage [34].

No clinical, gross pathological, or histopathological evidence of toxicity was reported during the two-week animal study. Wounds in both groups were at a comparable stage of granulation tissue healing, with no observed necrosis, mineralization, or fibrosis. Average scores for crust formation, epidermal hyperplasia, re-epithelialization, and hemorrhage were similar between PermeaDerm and Biobrane sites. Differences were noted primarily in inflammation and foreign debris, with lower average scores reported for PermeaDerm. These findings were attributed in part to a reduced foreign body response and fewer dressing fragments in the PermeaDerm-treated wounds [34].

These results suggested that PermeaDerm provided a favorable local tissue response while limiting fluid accumulation. In the full-thickness rat wound model, no fluid accumulation was observed in PermeaDerm-treated wounds, whereas fluid accumulation occurred in a subset of wounds covered with Biobrane. This finding supports the rationale for variable porosity as a design strategy to balance moisture control with exudate management [34].

Additional animal and cell-based experiments explored adherence and cellular response on PermeaDerm prototypes [34]. Human mesenchymal stromal cells and dermal fibroblasts incorporated tritiated thymidine when cultured on Biobrane, PermeaDerm B, and PermeaDerm T, as shown in Figure 1. Uptake on the AS and AS-2 formulations was approximately 1% of the Biobrane reference for both cell types. The demonstrated observation is therefore limited to markedly reduced DNA-synthesis signal under the reported culture conditions. Because concurrent measurements of cell viability, membrane integrity, metabolic activity, apoptosis, attachment, or absolute cell number were not reported, the experiment cannot determine whether the low signal resulted from reduced proliferation, cytotoxicity, impaired attachment, fewer cells, altered substrate accessibility, or another formulation-dependent effect. Accordingly, these data do not demonstrate an anti-scar mechanism or clinical anti-scar benefit and should be considered hypothesis-generating only.

Additional cell-culture experiments evaluated attachment, proliferation, and migration of human dermal fibroblasts on Biobrane, PermeaDerm B, and PermeaDerm T surfaces, as shown in Figure 2. Metabolically active human dermal fibroblasts were detected on all tested substrates, and proliferation and migration were reported under the study conditions. The measurements differed among formulations, with PermeaDerm T having the highest observed migration rate in this experimental system. These findings demonstrate formulation-associated differences in vitro but do not establish the responsible mechanism or predict comparative wound healing, scar formation, or other clinical outcomes [34].

### 2.8. Clinical Applications of PermeaDerm in Burns and Radiation-Induced Skin Injury

PermeaDerm was subsequently compared with Mepilex Ag in a randomized, single-blinded, multicenter trial of partial-thickness burns [35]. Sixty-seven patients were analyzed, including 35 assigned to PermeaDerm and 32 to Mepilex Ag; 42 were pediatric and 25 were adult patients. The study used parallel-group randomization, with blinded wound assessment planned on day 14. The groups did not differ significantly in complete re-epithelialization on day 14 (*p* = 0.36) or day 21 (*p* = 0.25), and Mepilex Ag showed a nonsignificant advantage on day 7 (*p* = 0.25). Among superficial partial-thickness injuries, the estimated odds ratio for healing with PermeaDerm at day 14 was 5.87, but the confidence interval was wide and crossed unity (95% CI 0.385–89.580; *p* = 0.203). PermeaDerm required fewer product changes (odds ratio 0.15, 95% CI 0.027–0.872; *p* = 0.034). Time to alternative therapy, pain, aesthetic outcomes, and adverse events, including infection, were similar between groups [35]. Thus, the trial showed broadly similar healing and safety outcomes, with fewer dressing changes in the PermeaDerm group; it did not demonstrate superiority for the primary healing outcome.

PermeaDerm has also been described in a single-patient report of moist desquamation of the neck after chemoradiotherapy for oropharyngeal squamous cell carcinoma [36]. In that case, the matrix adhered to the wound, allowed visual monitoring, and was associated with simpler wound care and less pain during healing. The report demonstrates feasibility in one radiation-induced wound, but it does not establish comparative efficacy or generalizability. Its relevance lies in showing how adherence, transparency, moisture management, and reduced dressing disruption may be useful in a setting other than thermal burns; confirmation will require prospective study.

More recently, PermeaDerm was evaluated against frozen human cadaveric allograft in a prospective, two-arm, randomized multicenter study of 40 patients treated at 11 U.S. burn centers. Both products were used for temporary coverage of excised wounds before definitive split-thickness skin grafting. Sponsor-reported results indicated a 70% reduction in product cost per percentage of total body surface area treated and a 95.7% reduction in preparation time with PermeaDerm. Application time, graft take, wound healing, and safety outcomes were reported as comparable between groups. Approximately 94% of PermeaDerm-treated patients achieved at least 95% graft take one week after autografting, all patients in both groups achieved at least 95% wound healing by eight weeks, and no adverse events were attributed to PermeaDerm. At final follow-up, all responding investigators and patients reported being satisfied or very satisfied. These findings are preliminary pending peer-reviewed publication of the complete methods and results [37].

The randomized multicenter trial provides comparative clinical evidence for PermeaDerm in adult and pediatric partial-thickness burns [35]. The radiation-injury publication is a separate single-patient feasibility report in a different indication [36]. A more recent multicenter randomized comparison with frozen human cadaveric allograft provides preliminary comparative evidence for wound temporization [37]. These sources should not be treated as equivalent: the published burn trial permits cautious comparative conclusions for its prespecified outcomes, the case report demonstrates individual feasibility only, and the newer allograft-comparison results remain sponsor-reported pending full peer-reviewed publication.

The material composition, immobilization approaches, and principal design characteristics of these technologies are summarized in Table 2, while their clinical applications, highest levels of supporting evidence, main findings, and key limitations are compared in Table 3.

## 3. Conclusions and Future Perspectives

Over five decades, collagen- and gelatin-containing technologies have evolved from relatively simple hemostatic materials and biologic coverings into increasingly engineered wound-contacting devices and regenerative biomaterials. The examples reviewed here illustrate that successful device performance depends not only on the biological material itself, but also on factors such as adherence, fluid management, porosity, transparency, mechanical integrity, manufacturability, and clinical usability. Collectively, these developments reflect a broader shift from viewing collagen and gelatin primarily as passive biomaterials toward using them as adaptable components within purpose-designed medical technologies.

The evidence supporting these technologies varies considerably in depth and quality. Biobrane and TransCyte were evaluated in comparative clinical studies, whereas the AWBAT evidence remains predominantly developmental, preclinical, and based on limited clinical observations. PermeaDerm is supported by preclinical studies, a published randomized multicenter trial, a separate single-patient report, and recently reported results from a second randomized multicenter comparison; however, the latter results await full peer-reviewed publication. As with many historical technology reviews, direct comparison across studies is complicated by differences in design, patient populations, outcome measures, and reporting practices. Consequently, some findings should be viewed as established clinical evidence, whereas others remain preliminary or hypothesis-generating.

Despite these limitations, the historical progression described here highlights several enduring themes. First, collagen and gelatin continue to offer a unique combination of biocompatibility, processability, and functional versatility that has enabled their use across a wide range of medical applications. Second, improvements in device performance have frequently resulted from advances in engineering design rather than from changes in the biological material alone. Finally, the field has consistently adapted collagen- and gelatin-based materials to emerging clinical needs, from temporary wound coverage and regenerative scaffolds to vaccine stabilization and biofabrication platforms.

Future progress will depend on combining advances in materials science with rigorous clinical evaluation. More comprehensive characterization of material composition, molecular-weight distribution, cross-linking, degradation behavior, moisture transport, and mechanical performance would improve comparison among studies and technologies. Independent, adequately powered clinical investigations are also needed to clarify the relative contributions of material properties and device design to patient outcomes. Such studies should evaluate not only healing endpoints, but also pain, infection, scar quality, dressing-change frequency, cost, and long-term clinical performance.

Collagen and gelatin remain among the most versatile biomaterials available for medical-device development. Their continued relevance is supported not only by their long history of clinical use, but also by ongoing innovations in recombinant production, engineered matrices, controlled-porosity systems, bioactive formulations, and regenerative medicine applications. The recent randomized comparison of PermeaDerm with cadaveric allograft brings this history full circle by revisiting the practical objective that initiated the skin-substitute development program in 1970. Preliminary results indicate comparable clinical outcomes with substantially reduced product cost and preparation time, although full peer-reviewed publication is needed before definitive conclusions can be drawn [37]. The next generation of collagen- and gelatin-based technologies will likely be defined by a balance among biological function, engineering design, manufacturing practicality, clinical evidence, and economic accessibility, building on lessons learned over more than five decades of development.

## Figures and Tables

**Figure 1:**
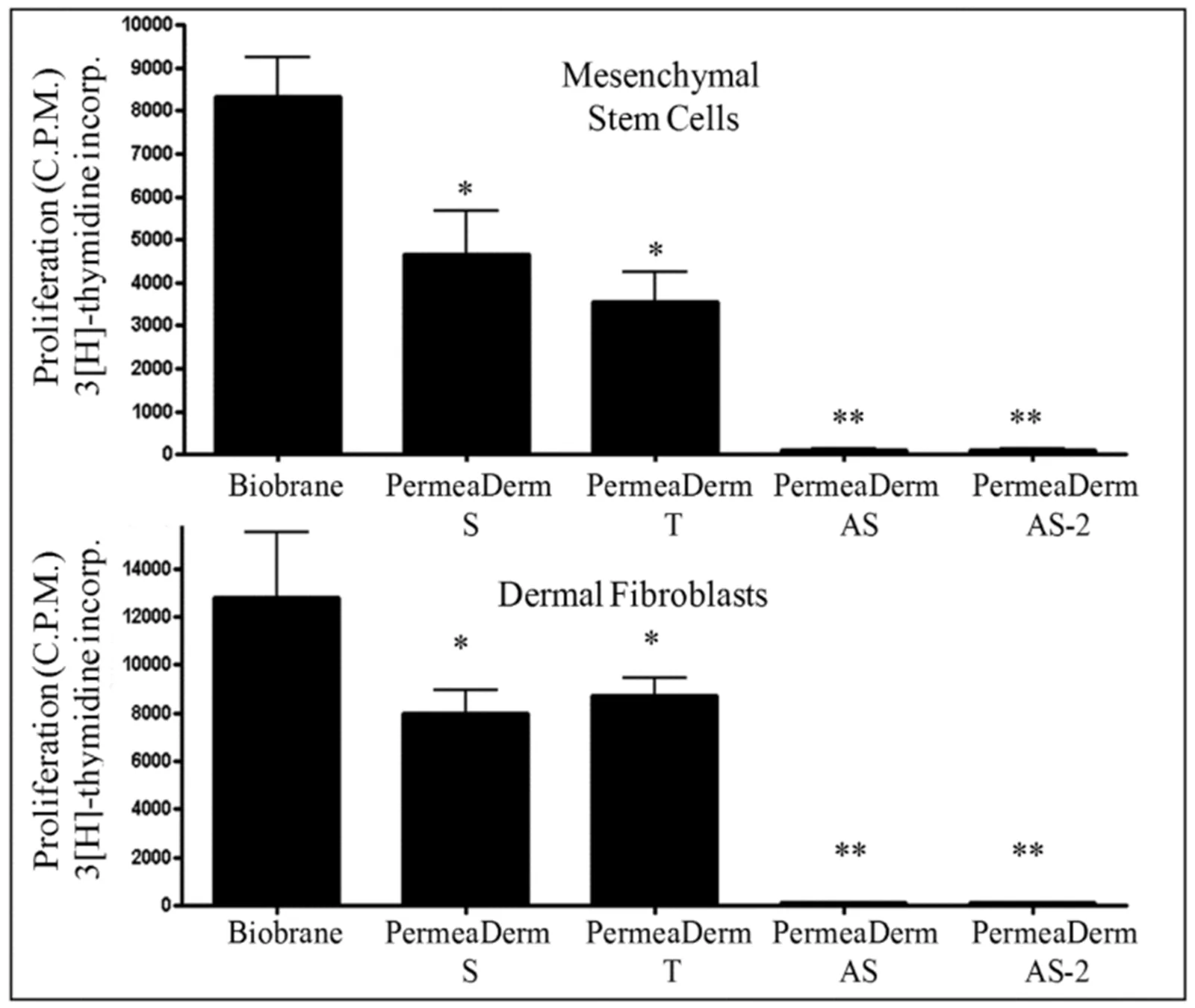
Tritiated-thymidine incorporation by human mesenchymal stromal cells (**upper panel**) and human dermal fibroblasts (**lower panel**) cultured on Biobrane and PermeaDerm prototype surfaces. Cells were cultured for 24 h in medium containing 10% fetal bovine serum before tritiated thymidine was added for a further 96 h as a measure of DNA synthesis. Results are reported relative to Biobrane. For mesenchymal stromal cells, relative uptake was 51% on PermeaDerm B, 41% on PermeaDerm T, and approximately 1% on PermeaDerm AS and AS-2. For dermal fibroblasts, relative uptake was 63.6% on PermeaDerm B, 69.7% on PermeaDerm T, and approximately 1% on PermeaDerm AS and AS-2 [34]. The assay demonstrates reduced thymidine incorporation on AS and AS-2 under these conditions but does not identify its cause. Because viability, membrane integrity, apoptosis, attachment, and absolute cell number were not measured concurrently, the low values cannot distinguish reduced proliferation from cytotoxicity, impaired attachment, reduced cell number, or another substrate-dependent effect. PermeaDerm S is equivalent to PermeaDerm. The data was analyzed by ANOVA using Prism software from Graphpad (* *p* < 0.05, ** *p* < 0.01).

**Figure 2:**
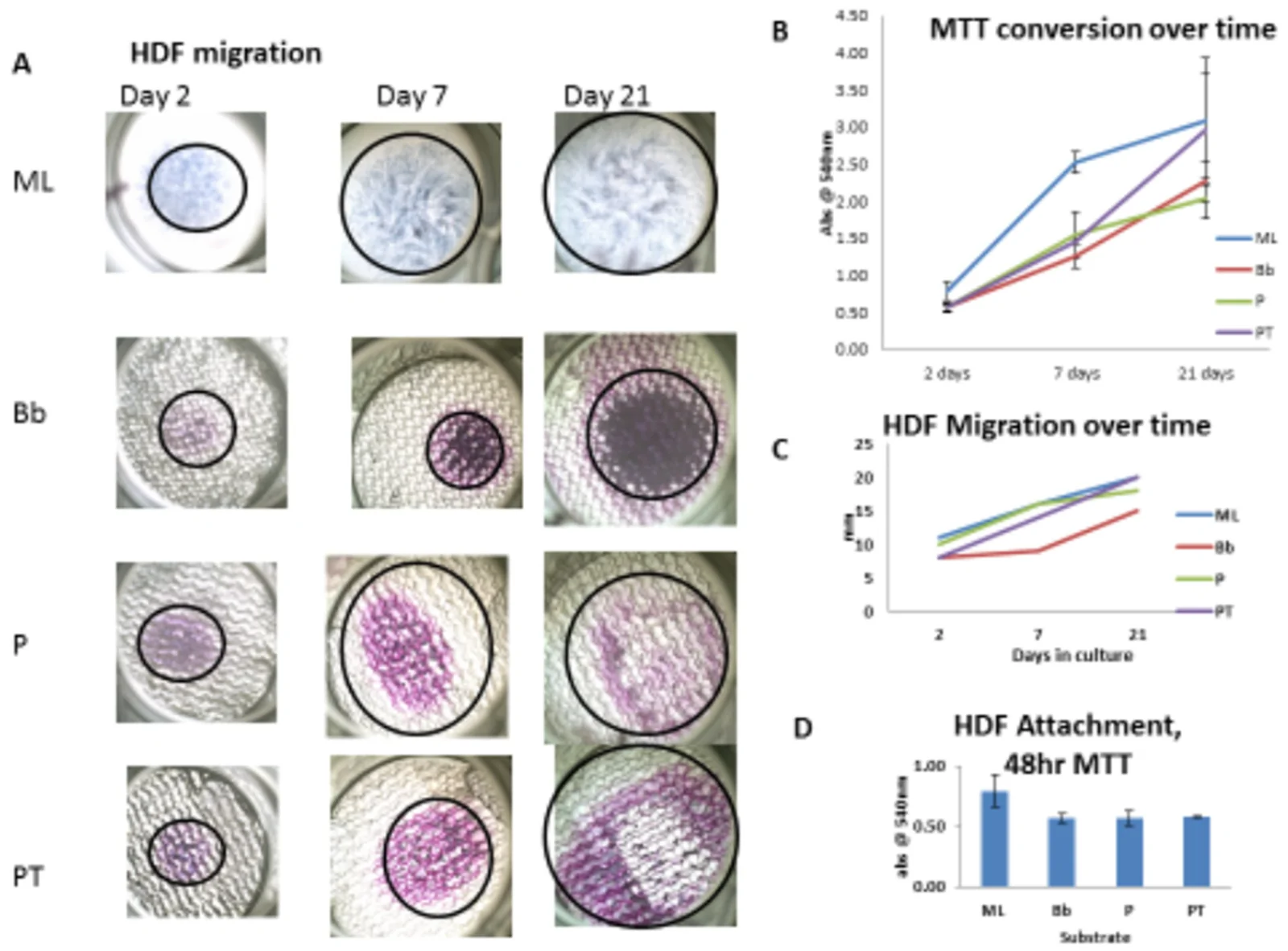
In vitro attachment, proliferation, and migration of human dermal fibroblasts on Biobrane and PermeaDerm prototype membranes. (**A**) Representative 100× bright-field images of MTT-stained cells on the tested substrates. HDF indicates human dermal fibroblast; ML, monolayer on polystyrene; Bb, Biobrane; P, PermeaDerm B; and PT, PermeaDerm T. (**B**) Reported HDF proliferation over time; ML showed the most rapid initial attachment and proliferation, and PT had the highest reported proliferation rate during the culture period. (**C**) Reported HDF migration rates differed among substrates, with the highest observed value for PT. (**D**) The 48 h MTT-conversion data were used as an indirect indicator of metabolically active cells associated with the substrate and were interpreted as reflecting initial attachment. ML served as the 100% reference, and the other tested materials were reported within 75% of ML binding. These in vitro observations do not establish a mechanism or comparative clinical benefit [34].

**Table 1:** Temporary skin-substitute formulations described in the developmental report [34].

Temporary Skin Substitute	Notes
Biobrane	Nylon mesh with gelatin
PermeaDerm B	Nylon mesh with silicone; approximately 24.5 slits/cm^2^ for burn applications
PermeaDerm C	Nylon mesh with silicone; approximately 48.1 slits/cm^2^ for chronic-wound applications
PermeaDerm	PermeaDerm B or C with a biological coating of HMW gelatin and aloe
PermeaDerm T	PermeaDerm B with standard coating and native collagen
PermeaDerm AS	PermeaDerm with anti-scar coating in silicone
PermeaDerm AS-2	PermeaDerm with anti-scar coating in silicone and biological layers

**Table 2:** Material composition and design characteristics of selected collagen- and gelatin-containing medical technologies.

Technology	Composition/Immobilization	Key Design Features	Persistence
Glutaraldehyde-fixed tissue heart valves	Bovine pericardial or porcine aortic tissue; glutaraldehyde fixation, with formaldehyde used alone or with glutaraldehyde [15].	Fixation improves strength, stability, and durability; comparable quantitative mechanical data were unavailable.	Long-term implant; calcification, deterioration, and immune-mediated failure remain possible [16].
Biobrane	Silicone film/nylon mesh with covalently coupled porcine-skin gelatin [17,23].	Flexible and transparent; pores permit fluid egress [23]. Standardized mechanical data were unavailable.	Temporary, externally removed covering; coating kinetics were not reported.
TransCyte	Biobrane-related mesh with fibroblast-deposited extracellular matrix; marketed acute-burn product was nonviable [27,28,29].	Flexible covering with preformed biological matrix; standardized mechanical and transport data were unavailable.	Temporary; separates or is removed after re-epithelialization. Matrix-degradation kinetics were not reported.
AWBAT	Silicone–nylon composite with biological coating; earlier covalent coupling agents were removed [33,34].	Modified structure and pores to improve conformity, adherence, fluid removal, and antimicrobial diffusion [33,34].	Temporary; degradation and coating-loss kinetics were not reported.
PermeaDerm	Nylon–silicone membrane with gelatin, aloe, native collagen, or investigational anti-scar formulations [34].	Flexible and transparent; variable slit density supports moisture and fluid control [34].	Temporary; degradation and coating-persistence kinetics were not reported.

**Table 3:** Clinical applications and supporting evidence for the selected technologies.

Technology/Indication	Highest Evidence Level	Main Findings	Key Limitation
Tissue heart valves Bioprosthetic valve replacement	Historical technical and mechanistic/clinical pathology evidence [15,16].	Fixation enabled durable implantation.	Antigenicity and late calcific failure remained; not directly comparable with wound studies.
Biobrane Temporary burn coverage	Within-patient clinical comparison and retrospective cohort, supported by animal studies [18,19,20,21,22,23,24,25,26].	Comparable with allograft; retrospective data suggested shorter procedures and lower reported costs.	Study design, era, and patient selection limit generalizability.
TransCyte Partial- and full-thickness burns	Small prospective randomized trials plus noncomparative observations [27,28,29,30,31,32].	Selected comparisons favored healing time, dressing changes, scar outcomes, or pain.	Small cohorts and study-era practices limit inference.
AWBAT Burn and donor-site coverage	Developmental/preclinical evidence and limited donor-site observations [33,34].	Limited experience reported feasibility, no infections, and shorter healing than Acticoat.	Small sample and limited reporting do not establish comparative efficacy.
PermeaDerm Burn temporization; partial-thickness burns; radiation-injury feasibility	Randomized multicenter burn trial, randomized multicenter comparison study, single-patient radiation report, and preclinical studies [34,35,36,37].	Similar healing and safety versus Mepilex Ag, with fewer changes [35]; one-patient radiation feasibility [36]; preliminary allograft comparison reported 70% lower product cost, 95.7% less preparation time, and comparable clinical outcomes [37].	No superiority for the primary healing outcome in the published trial; case report is not generalizable; allograft-comparison results await peer-reviewed publication.

## Data Availability

No new data were created or analyzed in this study. Data sharing is not applicable to this article.

## Abbreviations

The following abbreviations are used in this manuscript:

AWBAT	Advanced Wound Biosynthetic Alternative Tissue
GA	Glutaraldehyde
FA	Formaldehyde
FDA	U.S. Food and Drug Administration
HMW	High molecular weight
HDF	Human dermal fibroblast
